# Vascular endothelial growth factor (VEGF) upregulates BCL-2 and inhibits apoptosis in human and murine mammary adenocarcinoma cells

**DOI:** 10.1054/bjoc.2001.1876

**Published:** 2001-07

**Authors:** G P Pidgeon, M P Barr, J H Harmey, D A Foley, D J Bouchier-Hayes

**Affiliations:** Department of Surgery, Royal College of Surgeons in Ireland, Beaumont Hospital, Beaumont, Dublin 9, Ireland

**Keywords:** vascular endothelial growth factor, Bcl-2, apoptosis

## Abstract

Tumour progression is regulated by the balance of proliferation and apoptosis in the tumour cell population. To date, the role of vascular endothelial growth factor (VEGF) in tumour growth has been attributed to the induction of angiogenesis. VEGF has been shown to be a survival factor for endothelial cells, preventing apoptosis by inducing Bcl-2 expression. In both murine (4T1) and human (MDA-MB-231) metastatic mammary carcinoma cell lines, we found that VEGF upregulated Bcl-2 expression and anti-VEGF antibodies reduced Bcl-2 expression. These alterations in Bcl-2 expression were reflected by the levels of tumour cell apoptosis. VEGF resulted in reduced tumour cell apoptosis, whereas its inhibition with anti-VEGF neutralizing antibodies induced apoptosis directly in tumour cells. Therefore, in addition to its role in angiogenesis and vessel permeability, VEGF acts as a survival factor for tumour cells, inducing Bcl-2 expression and inhibiting tumour cell apoptosis. © 2001 Cancer Research Campaign http://www.bjcancer.com


British Journal of Cancer (2001) **85**(2), 273–278
© 2001 Cancer Research Campaign
doi: 10.1054/ bjoc.2001.1876, available online at http://www.idealibrary.com on IDEAL®

http://www.bjcancer.com

### Vascular endothelial growth factor (VEGF) upregulates BCL-2 and inhibits apoptosis in human and murine mammary adenocarcinoma cells

**GP Pidgeon, MP Barr, JH Harmey, DA Foley and DJ Bouchier-Hayes**

Royal College of Surgeons in Ireland, Department of Surgery, Beaumont Hospital, Beaumont, Dublin 9, Ireland

**Summary** Tumour progression is regulated by the balance of proliferation and apoptosis in the tumour cell population. To date, the role of vascular endothelial growth factor (VEGF) in tumour growth has been attributed to the induction of angiogenesis. VEGF has been shown to be a survival factor for endothelial cells, preventing apoptosis by inducing Bcl-2 expression. In both murine (4T1) and human (MDA-MB-231) metastatic mammary carcinoma cell lines, we found that VEGF upregulated Bcl-2 expression and anti-VEGF antibodies reduced Bcl-2 expression. These alterations in Bcl-2 expression were reflected by the levels of tumour cell apoptosis. VEGF resulted in reduced tumour cell apoptosis, whereas its inhibition with anti-VEGF neutralizing antibodies induced apoptosis directly in tumour cells. Therefore, in addition to its role in angiogenesis and vessel permeability, VEGF acts as a survival factor for tumour cells, inducing Bcl-2 expression and inhibiting tumour cell apoptosis. © 2001 Cancer Research Campaign http://www.bjcancer.com

**Keywords**: vascular endothelial growth factor; Bcl-2; apoptosis

Angiogenesis, the development of new blood vessels, is an essential requirement for both primary and metastatic tumour growth. In the absence of angiogenesis tumours cannot grow beyond 1–2 $mm^3$ in size (Gimbrone et al, 1972). The ability of tumours to stimulate neovascularization is governed by the net balance of angiogenic stimulators and inhibitors (Hanahan and Folkman, 1996). Tumours can secrete or mobilize a variety of angiogenic factors that tip the balance in favour of angiogenesis. Vascular endothelial growth factor (VEGF) also known as vascular permeability factor, VPF, is a potent angiogenic factor and endothelial cell mitogen (Jakeman et al, 1992). Produced by a variety of cell types and tumours, its major action has been attributed to the neovascularization of tumours. By increasing vascular permeability (Senger et al, 1983), resulting in the leaky vasculature characteristic of tumour angiogenesis, VEGF facilitates extravasation of tumour cells and the new vessels induced by VEGF provide an exit route for metastatic tumour cells.

Under normal physiological conditions, a delicate balance between cell proliferation and cell death ensures that the overall numbers of cells are maintained within an appropriate range. The selective process whereby cells are discretely removed from populations without affecting surrounding cells is termed apoptosis or 'programmed cell death'. Disturbances in this process may confer a growth advantage to neoplastic tissues (Thompson, 1995). The Bcl-2 family of apoptosis-regulating proteins function to either promote or suppress cell death (Oltvai et al, 1993). Increased expression of the anti-apoptotic protein, Bcl-2, has been reported in many tumours and paradoxically, increased expression of Bcl-2 is a good prognostic indicator in some cancers (Krawjewski et al, 1999). Conversely, overexpression of Bcl-$X_L$ reduced chemotherapy-induced apoptosis of mammary tumours in a murine model (Liu et al, 1999).

*Received 25 August 2000*
*Revised 14 March 2001*
*Accepted 15 March 2001*

*Correspondence to:* JH Harmey

VEGF has been shown to protect endothelial cells from apoptosis induced by growth factor withdrawal, and this protection is associated with increased Bcl-2 expression (Nor et al, 1999). Furthermore, VEGF has been shown to protect non-endothelial cells, namely leukaemia cells and haematopoietic stem cells, from radiation-induced apoptosis (Katoh et al, 1998) whereas neutralizing antibody to VEGF increased the efficacy of ionizing radiation in tumour-bearing mice (Gorski et al, 1999).

Endotoxin/lipopolysaccharide (LPS), a cell wall constituent of Gram-negative bacteria, is released during growth or lysis of bacteria and acts as a potent inflammatory stimulus, eliciting a range of cytokines. In an experimental metastasis model we previously demonstrated that intra-peritoneal injection of LPS resulted in elevated circulating VEGF and decreased apoptosis within lung metastases relative to control mice (Pidgeon et al, 1999). In vitro, LPS directly increased VEGF expression by 4T1 murine mammary adenocarcinoma cells and increased VEGF expression by human pulp cells exposed to LPS has also been reported (Matsushita et al, 1999; Pidgeon et al, 1999).

We hypothesized that VEGF acts as a survival factor for tumour cells as well as endothelial cells, inhibiting tumour cell apoptosis by inducing Bcl-2 expression. Bcl-2 expression and apoptosis in human MDA-MB-231 and murine 4T1 cells in response to VEGF, LPS (to stimulate VEGF) and neutalizing antibodies to VEGF (to block endogenous or exogenous VEGF) were evaluated.

### MATERIALS AND METHODS

### Cell culture

The spontaneously metastasizing murine mammary adenocarcinoma cell line 4T1 was generously provided by Mr E Coveny

(Waterford Regional Hospital, Co Waterford, Ireland). The human mammary metastatic cell line MDA-MB-231 was originally obtained from the ATCC. All cell culture reagents were obtained from GIBCO-BRL (Paisley, UK). The 4T1 cells were maintained in a humidified atmosphere of 5% $CO_2$ in air at 37°C in RPMI, supplemented with 10% heat-inactivated fetal calf serum, 100 U $ml^{-1}$ penicillin and 100 mg $ml^{-1}$ streptomycin sulfate. The MDA-MB-231 cells were maintained in sealed flasks at 37°C in L-15, supplemented with 10% heat-inactivated fetal calf serum, 100 U $ml^{-1}$ penicillin and 100 μg $ml^{-1}$ streptomycin sulfate. In-vitro experiments were performed when cells were approximately 80% confluent.

### Treatment of cells

For Western analysis, cells were seeded at $5 \times 10^5$ cells/75 $cm^3$ flask. Cells were allowed to recover for 18–24 h and then incubated with either 10 μg $ml^{-1}$ LPS (to induce VEGF expression), 100 ng $ml^{-1}$ recombinant VEGF (R&D Systems, UK), 10 μg $ml^{-1}$ LPS with 1 μg $ml^{-1}$ neutralizing VEGF pAb (R&D Systems, UK) or VEGF pAb alone for 18 h.

### Western analysis

Culture fluid was aspirated and cells pelleted at 300 ***g*** for 10 min and combined with adherent cells which were washed twice with PBS and lysed on ice for 30 min in 1 ml lysis buffer (5 mM Tris-HCL, pH 7.4, 150 mM NaCl, 5 mM EDTA, 0.5 % Triton X-100, 0.5 % SDS. 0.005% deoxycholate) 1 mM phenylmethylsulfonylflouride (PMSF). Lysates were passed through a 20 G needle, boiled for 10 min and centrifuged at 12 000 ***g*** for 15 min at 4°C. Total protein concentration in cleared lysate was determined by the BCA assay according to manufacturers instructions (Pierce Chemical Co, Illinois). 50 μg protein was separated on 12% denaturing polyacrylamide gels and transferred to nitrocellulose membranes by electroblotting. Membranes were blocked for 1 hour with Tris-buffered saline containing 0.05% Tween 20 (TBST) and 5% non-fat milk protein and incubated for 90 min with anti-Bcl-2 or anti-β-actin antibodies diluted 1:200 in TBST containing 5% non-fat dried milk (Santa Cruz Biotech, CA, US). The membrane was washed 3 times in TBST, incubated for 90 min with horse-radish peroxidase conjugated goat anti-rabbit IgG (1:2000 in TBST, Dako), and washed 6 times in TBST. Bound antibody complexes were visualized using enhanced chemiluminescence (Pierce Chemical Co, IL).

Antibody binding was quantitated densitometrically (Vilber-Lourmat, Marne La Vallee, France). The amount of Bcl-2 staining was normalized against β-actin staining by calculating a bcl-2/β-action ratio for each sample.

### TUNEL staining for apoptosis

Cells were seeded on glass (MDA-MB-231) or plastic (4T1) culture chamber slides (Falcon, UK) at a concentration of $5 \times 10^4$ cells/chamber, allowed to recover overnight and then treated for 18 h with the same treatment protocol outlined previously. Culture medium was supplemented with 1% FCS (as opposed to 10%) to stimulate apoptosis by growth factor withdrawal. Culture chamber slides were fixed in 100% acetone for 5 min and apoptotic cells were stained using the in situ cell death detection assay (Boehringer Mannheim, UK) for the demonstration of DNA fragmentation. Sections were counterstained with haematoxylin. Apoptotic cells stained brown and an apoptotic index was estimated under a light microscope at × 400 magnification using a 1 mm grid by 2 independent observers (GP and MB). A minimum of 3000 cells (6 high-power fields) were counted over 3 separate chambers of each treatment group.

### Statistical analysis

Statistical comparison between study groups was carried out using ANOVA with Scheffe post-hoc correction in DataDesk 4.1. Results are expressed as mean ± standard error mean (SEM). Data were taken as significant where $P < 0.05$.

### RESULTS

We previously examined the effect of LPS exposure on tumour growth in a murine model where experimental lung metastases were established by tail vein injection of 4T1 tumour cells. LPS exposure resulted in increased circulating VEGF and decreased apoptosis within lung tumour nodules (Pidgeon et al, 1999). In this study we examined the effect of VEGF on tumour cell apoptosis using LPS to induce VEGF expression and a neutralizing antibody to block VEGF activity. We previously showed that 10 μg $ml^{-1}$ LPS for 18 h increased VEGF expression by 4T1 cells from a basal level of 59.1 ± 1.87 to 117.22 ± 11.89 pg VEGF $\mu g^{-1}$ total cell protein ($P < 0.01$) (Pidgeon et al, 1999). Basal VEGF expression by MDA-MB-231 cells was less than 4T1 cells but was also significantly increased following treatment with 10 μg $ml^{-1}$ LPS for 18 h (from 20.56 ± 1.51 to 28.32 ± 2.05 pg VEGF $\mu g^{-1}$ total cell protein ($P < 0.05$)).

### Effect of VEGF, LPS and anti-VEGF antibodies on Bcl-2 protein expression

The level of Bcl-2 protein expression was examined by Western blot analysis following treatment with LPS, VEGF and/or anti-VEGF antibodies. The neutralization $dose_{50}$, that is the dose of antibody required to yield one half maximal inhibition, ($ND_{50}$) for anti-hVEGF was 5–10 μg $ml^{-1}$ to neutralize 10 ng $ml^{-1}$ rhVEGF and for anti-mVEGF the $ND_{50}$ was 0.05–0.15 μg $ml^{-1}$ to neutralize 10 ng $ml^{-1}$ rmVEGF. Western blot analysis showed that VEGF (100 ng $ml^{-1}$) or LPS (10 μg $ml^{-1}$) resulted in increased Bcl-2 expression in both 4T1 and MDA-MB-231 cells relative to controls (Figure 1A and Figure 2A, respectively). Densitometry confirmed that relative Bcl-2 expression by 4T1 and MDA-MB-231 cells was significantly increased following treatment with either LPS (4T1, 140 ± 7.5%; MDA-MB-231, 138 ± 13.4%) or VEGF (4T1, 136 ± 7.6%; MDA-MB-231, 166 ± 31.2%) compared to controls ($P < 0.02$, Figure 1B and Figure 2B, respectively). The addition of a neutralizing anti-VEGF antibody (1 μg $ml^{-1}$) decreased Bcl-2 protein expression in both cell lines (4T1, 46 ± 10.2%; MDA-MB-231, 18 ± 3.5%). In this case the antibody is blocking basal VEGF expressed by the tumour cells. Densitometric analysis illustrated that this blockade of endogenous VEGF was significant relative to controls ($P < 0.03$). Anti-VEGF antibodies blocked LPS induced Bcl-2 expression resulting in a significant decrease in Bcl-2 protein levels relative to controls (4T1, 51 ± 11.1%; MDA-MB-231, 48 ± 6.6%) ($P < 0.03$). However, in the case of MDA-MB-231 cells, in the presence of LPS, VEGF antibody did not inhibit Bcl-2 expression as much as VEGF antibody alone. LPS is a non-specific stimulus and may

 © 2001 Cancer Research Campaign

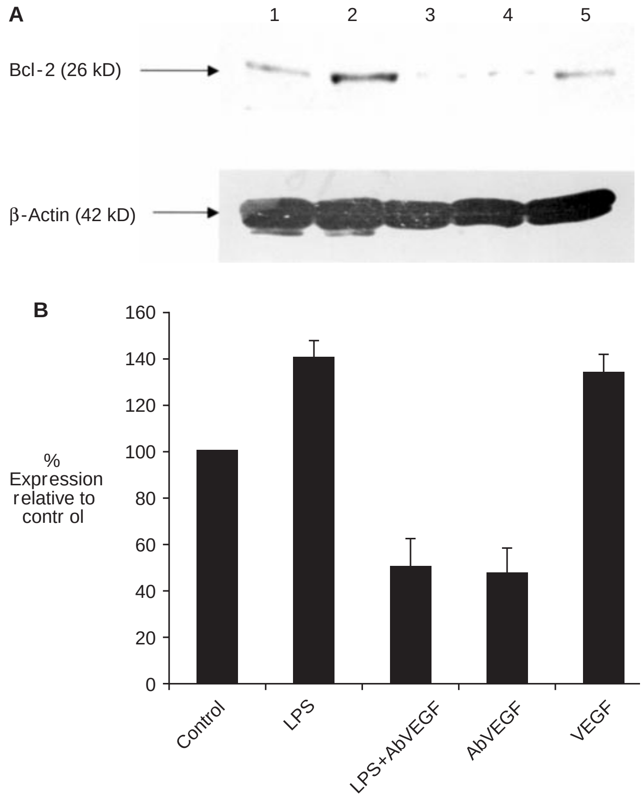

**Figure 1** (**A**) Bcl-2 protein expression in 4T1 cells. Bcl-2 expression was examined by Western blot. β-actin expression was also examined to control for loading differences. Untreated cells are shown in lane 1. Treatment with 10 μg ml$^{-1}$ LPS (lane 2) or 100 ng ml$^{-1}$ VEGF (lane 5) resulted in increased Bcl-2 expression relative to control cells. Neutralizing antibody to VEGF alone reduced basal Bcl-2 expression relative to controls (lane 4) and blocked LPS-induced Bcl-2 (lane 3). Western blot shown is representative of 4 independent experiments. (**B**) Densitometric analysis of Bcl-2 protein relative to β-actin. Values are expressed as expression relative to control cells (100%)

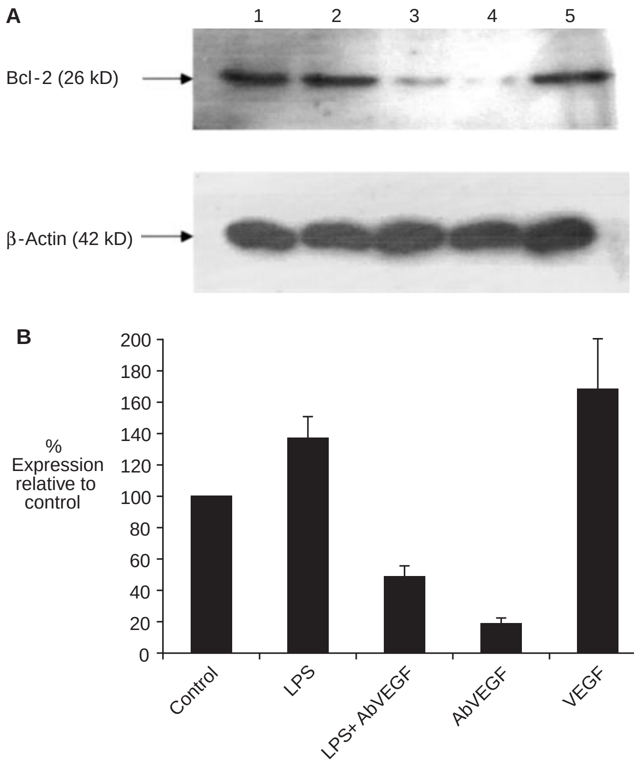

**Figure 2** (**A**) Bcl-2 protein expression in MDA-MB-231 cells. Bcl-2 expression was examined by Western blot. β-actin expression was also examined to control for loading differences. Untreated cells are shown in lane 1. Treatment with 10 μg ml$^{-1}$ LPS (lane 2) or 100 ng ml$^{-1}$ VEGF (lane 5) resulted in increased Bcl-2 expression relative to control cells. Neutralizing antibody to VEGF alone reduced basal Bcl2 expression relative to controls (lane 4) and blocked LPS-induced Bcl-2 (lane 3). Western blot shown is representative of 3 independent experiments. (**B**) Densitometric analysis of Bcl-2 protein relative to β-actin. Values are expressed as expression relative to control cells (100%)

induce other cytokines and growth factors in addition to VEGF. These data show that anti-VEGF antibodies reduce Bcl-2 expression in both 4T1 and MDA-MB-231 cells. Furthermore both LPS and VEGF treatment increase Bcl-2 expression and anti-VEGF blocks LPS-induced Bcl-2 expression.

### Effect of VEGF, LPS and anti-VEGF antibodies on tumour cell apoptosis

Having demonstrated that LPS and VEGF upregulate expression of the anti-apoptotic protein, Bcl-2, in human and murine tumour

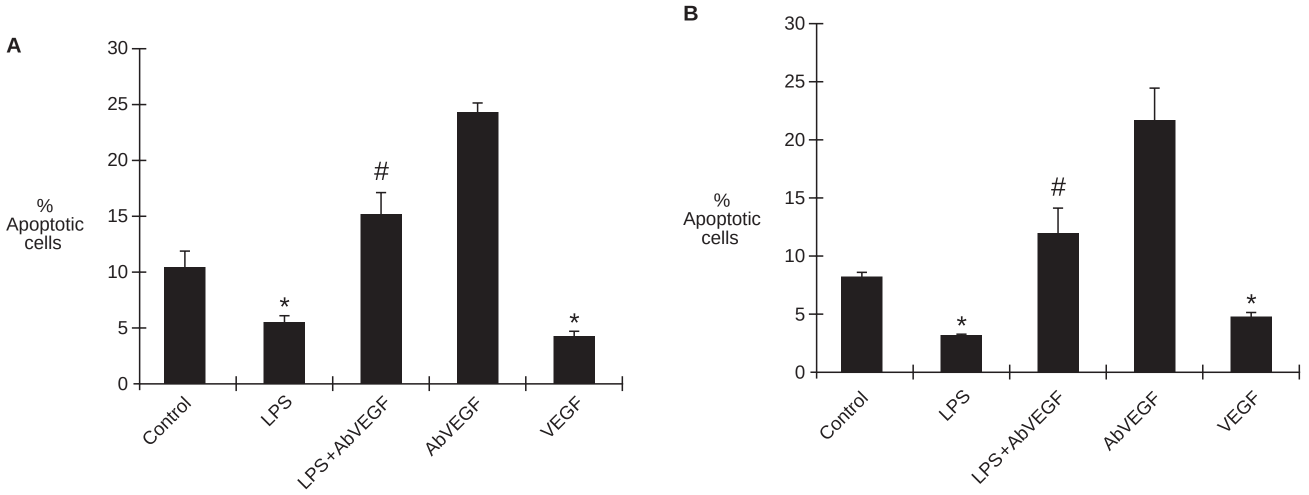

**Figure 3** *Continued*

*© 2001 Cancer Research Campaign*

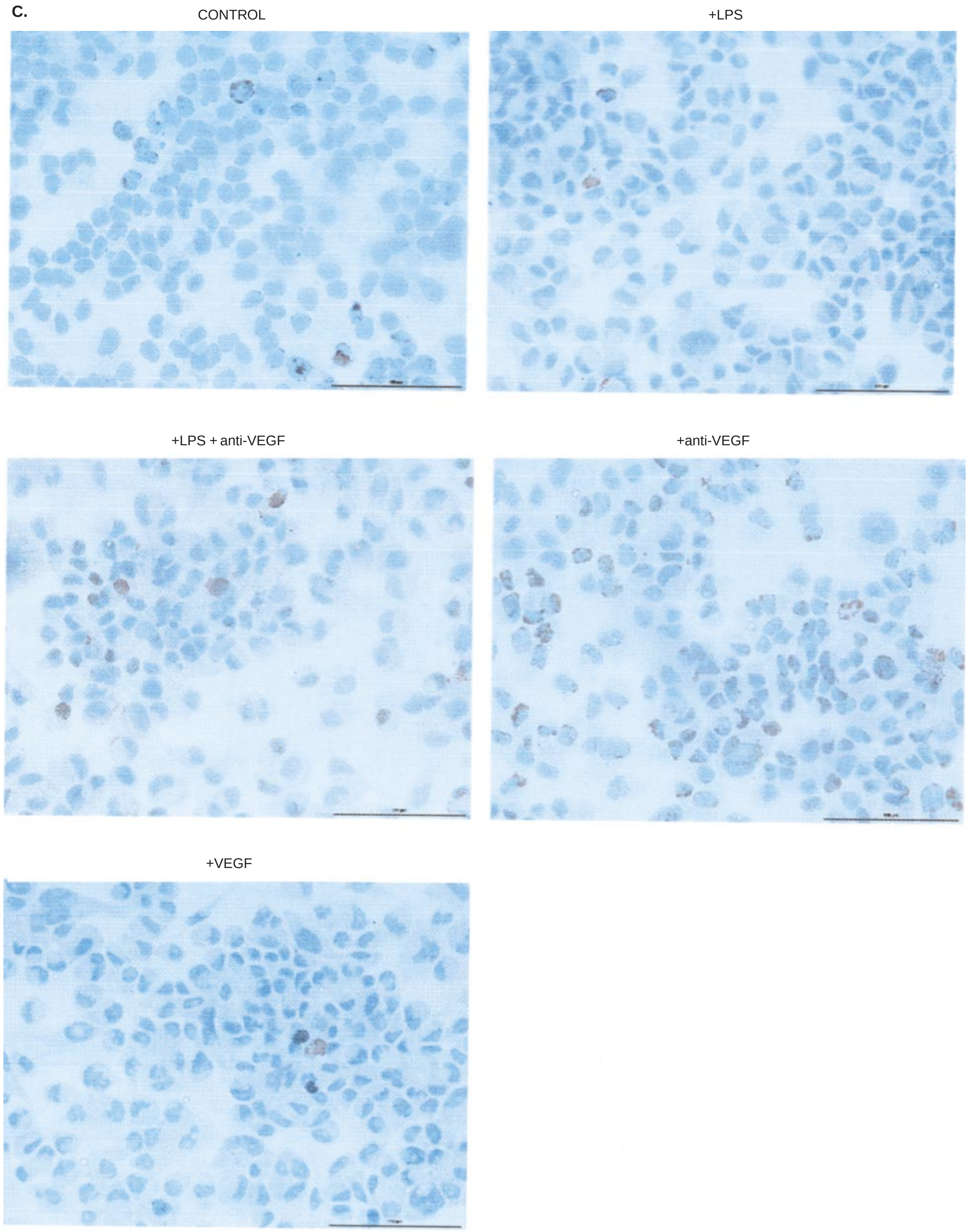

**Figure 3** (**A**) Levels of apoptosis in 4T1 cells were examined in chamber slides by TUNEL staining ($n = 3$). Apoptosis was induced to a level of 10.04% in control cells by growth factor depletion. Treatment with 10 μg $ml^{-1}$ LPS or 100 ng $ml^{-1}$ VEGF resulted in a significant reduction in the percentage of cells undergoing apoptosis. Antibody to VEGF alone significantly increased apoptosis relative to controls ($P < 0.03$) and inhibited LPS-mediated inhibition of apoptosis. *$P < 0.05$ vs control; # $P < 0.05$ vs LPS. (**B**) Levels of apoptosis in MDA-MB-231 cells were examined ($n = 3$). Apoptosis was induced to a level of 8.4% in control cells by growth factor depletion. Treatment with 10 μg $ml^{-1}$ LPS or 100 ng $ml^{-1}$ VEGF resulted in a significant reduction in the percentage of cells undergoing apoptosis. Antibody to VEGF alone significantly increased apoptosis relative to controls ($P < 0.03$) and inhibited LPS-mediated inhibition of apoptosis. *$P < 0.05$ vs control; # $P < 0.05$ vs LPS. (**C**) Representative MDA-MB-231 cells from each treatment group following TUNEL staining. Bar represents 100 μm

*© 2001 Cancer Research Campaign*

cells and that this expression can be blocked with anti-VEGF antibodies, we studied their effect on tumour cell apoptosis directly by TUNEL staining on culture chamber slides. Due to the low basal rate of apoptosis in tumour cells, apoptosis was induced to a level of 10.33 ± 1.41% in 4T1 and 8.4 ± 0.47% in MDA-MB-231 cells by growth factor withdrawal (Figure 3A and B, respectively). Treatment with LPS (4T1, 5.39 ± 0.66%; MDA-MB-231, 3.4 ± 0.18%) or VEGF (4T1, 4.07 ± 0.44%; MDA-MB-231, 4.2 ± 0.39%) resulted in a significant decrease in the rate of apoptosis compared to untreated cells (4T1, 10.33 ± 1.41%; MDA-MB-231, 8.4 ± 0.47% $P < 0.05$, Figure 3A and B, respectively). Treatment of cells with anti-VEGF antibodies resulted in a significant increase in the level of apoptosis in both cell lines (24.2 ± 0.90%, $P < 0.01$ in 4T1 cells and 22 ± 2.84%, $P < 0.02$ in MDA-MB-231 cells). In both cell lines, anti-VEGF antibodies prevented LPS-mediated inhibition of apoptosis compared to cells treated with LPS alone (15.12 ± 1.98% vs 5.39 ± 0.66%, $P < 0.01$ in 4T1) and (11.8 ± 2.23% vs 3.4 ± 0.18%, $P < 0.005$ in MDA-MB-231). Typical stained MDA-MB-231 cells from each treatment group are shown in Figure 3C. Increased numbers of TUNEL-positive cells are clearly visible in samples treated with anti-VEGF antibody relative to untreated controls. Blocking either endogenous VEGF produced by tumour cells or exogenous VEGF (in this case induced by LPS exposure) leads to increased apoptosis, indicating that VEGF increases tumour cell survival by inhibiting apoptosis.

### DISCUSSION

The potent inflammatory mediator endotoxin/lipopolysaccharide (LPS) has been shown to be angiogenic in a number of experimental systems (Li et al, 1991; Mattsby-Balzer et al, 1994; Kenyon et al, 1996). However, the mechanism of its angiogenic activity has to date, been unknown. We previously demonstrated that LPS increases VEGF expression by human and murine metastatic breast carcinoma cells (Pidgeon et al, 1999). As LPS also increased VEGF expression by pulp cells (Matsushita et al, 1999), increased VEGF expression may account for the angiogenic activity of LPS previously reported.

VEGF has previously been shown to induce the expression of the anti-apoptotic protein Bcl-2 in endothelial cells, thereby prolonging their survival (Nor et al, 1999). We have demonstrated that both endogenous and exogenous VEGF (rVEGF) increased Bcl-2 expression with a concomitant inhibition of apoptosis in murine and human metastatic tumour cells. Neutralizing antibody to VEGF, reduced Bcl-2 expression and increased apoptosis in these cells. LPS induced Bcl-2 expression and inhibition of tumour cell apoptosis was abolished by anti-VEGF antibodies, demonstrating that these effects of LPS were mediated by VEGF.

Typically VEGF has been considered an endothelial cell specific survival factor. However, we demonstrate that VEGF is a survival factor for both murine 4T1 and human MDA-MB-231 tumour cells, preventing tumour cell apoptosis. The signalling pathway through which VEGF acts on tumour cells remains to be clarified. However, recently a novel third receptor, neuropilin 1, specific for VEGF165, has been identified on a number of tumour cells including MDA-MB-231 (Soker et al, 1998). Western blot analysis has failed to identify either KDR/Flk-1 or Flt1, the VEGF receptors expressed by endothelial cells, in 4T1 or MDA-MB-231 cells (data not shown). A role for VEGF in preventing tumour cell apoptosis is further supported by recent reports that overexpression of soluble neuropilin 1(sNRP1), which prevents VEGF165 binding to cell surface receptors, in tumour cells was associated with increased tumour cell apoptosis in vitro (Gagnon et al, 2000). These authors also demonstrated that sNRP1 prevented VEGF165 binding to rat prostate carcinoma cells, although the consequences of VEGF binding to these tumour cells was unknown. VEGF has been shown to upregulate the expression of the KDR receptor on endothelial cells which may be a positive feedback mechanism for VEGF action (Shen et al, 1998). Thus strategies that block VEGF activity may reduce VEGF receptor expression. Decreased VEGF receptor expression may therefore lead to an overall reduction in VEGF expression.

Anti-angiogenic therapy has received a lot of attention in the last decade. A number of studies have shown that anti-angiogenic strategies result in increased tumour cell apoptosis (Holmgren et al, 1995) an effect that to date, has been attributed to blood vessel regression. However, the angiogenic factor VEGF inhibits radiation-induced tumour cell apoptosis and chemotherapy-induced apoptosis of haematopoietic cells, the latter effect being achieved by the induction of MCL1, a member of the bcl-2 family (Katoh et al, 1998; Gorski et al, 1999). These observations can be explained in terms of our findings, namely that VEGF acts directly on tumour cells to prevent apoptosis. Our study suggests that anti-angiogenics, in particular those directed against VEGF, may have multiple anti-tumour effects. Firstly, they prevent endothelial cell growth inducing apoptosis resulting in regression of tumour vessels. Secondly, by blocking or reducing angiogenic molecules they may have a direct anti-tumour effect by increasing tumour cell apoptosis.

In conclusion, we have demonstrated that VEGF confers a survival advantage on tumour cells by upregulating Bcl-2 expression and inhibition of tumour cell apoptosis. Furthermore, neutralizing antibody to VEGF increased the efficacy of ionizing radiation in tumour-bearing mice (Gorski et al, 1999). Thus anti-VEGF strategies, as part of combination therapy, should improve the efficacy of conventional treatments aimed at inducing tumour cell apoptosis, such as chemotherapy and/or radiation therapy.

### ACKNOWLEDGEMENTS

This work was supported by grants from the Health Research Board RP182/2000, the Royal College of Surgeons in Ireland Research Committee and the Beaumont Hospital Cancer Research and Development Trust.

### REFERENCES

Gagnon ML, Bielenberg DR, Gechtman Z, Miao H-Q, Takashima S, Soker S and Klagsbrun M (2000) Identification of a natural soluble neuropilin-1 that binds vascular endothelial growth factor: *In vivo* expression and anti-tumour activity. *Proc Natl Acad Sci USA* **97**: 2573–2578

Gimbrone MA, Leapman SB, Cotran RS and Folkman J (1972) Tumour dormancy *in vivo* by prevention of neovascularisation. *J Exp Med* **136**: 261–276

Gorski DH, Beckett MA, Jaskowiak NT, Calvin DP, Mauceri HJ, Salloum RB, Seetharam S, Koons A, Hari DM, Kufe DW and Weichselbaum RR (1999) Blockade of the vascular endothelial growth factor stress response increases the antitumour effects of ionizing radiation. *Cancer Res* **59**: 3374–3378

Hanahan D and Folkman J (1996) Patterns and emerging mechanisms of the angiogenic switch during tumourigenesis. *Cell* **86**: 353–364

Holmgren L, O'Reilly MS and Folkman J (1995) Dormancy of micrometastases: balanced proliferation and apoptosis in the presence of angiogenesis supression. *Nature Medicine* **1**: 149–153

© 2001 Cancer Research Campaign 

Jakeman LB, Winer J, Bennett GL, Alter CA and Ferrara N (1992) Binding sites for vascular endothelial growth factor are localised on endothelial cells in adult rat tissue. *J Clin Invest* **89**: 244–253

Katoh O, Takahashi T, Oguri T, Kuramoto K, Mihara K, Kobayashi M, Hirata S and Watanabe H (1998) Vascular endothelial growth factor inhibits apoptotic death in hematopoietic cells after exposure to chemotherapeutic drugs by inducing MCL1 acting as an antiapoptotic factor. *Cancer Res* **58**: 5565–5569

Kenyon BM, Voest EE, Chen CC, Flynn E, Folkman J and D'Amato R (1996) A model of angiogenesis in the mouse cornea. *Invest Opthal and Vis Sci* **37**: 1625–1635

Krajewski S, Krajewska M, Turner BC, Pratt C, Howard B, Zapata JM, Frenkel V, Robertson S, Ionov Y, Yamamoto H, Perucho M, Takayama S and Reed JC (1999) Prognostic significance of apoptosis regulators in breast cancers. *Endocrine-Related Cancer* **6**: 29–40

Li WW, Grayson G, Folkman J and D'Amore PA (1991) Sustained- release endotoxin. A model for inducing corneal neovascularization. *Invest Opthal and Vis Sci* **32**: 2906–2911

Liu R, Page C, Beidler DR, Wicha MS and Nunez G (1999) Overexpression of Bcl-$X_L$ promotes chemotherapy resistance of mammary tumours in a syngeneic mouse model. *Amer J Pathol* **155**: 1861–1867

Matsushita K, Motani R, Sakuta T, Nagaoka S, Matsuyama T, Abeyama K, Maruyama I, Takada H and Torii M (1999) Lipopolysaccharide enhances the production of vascular endothelial growth factor by human pulp cells in culture. *Infection and Immunity* **67**: 1633–1639

Mattsby-Baltzer I, Jakobsson A, Sörbo J and Norrby K (1994) Endotoxin is angiogenic. *Int J Exp Path* **75**: 191–196

Nor JE, Christensen J, Mooney DJ and Polverini PJ (1999) Vascular endothelial growth factor (VEGF)-mediated angiogenesis is associated with enhanced endothelial cell survival and induction of Bcl-2 expression. *Amer J Pathol* **154**: 375–384

Oltvai ZN, Milliman CL and Korsmeyer SJ (1993) Bcl-2 heterodimers *in vitro* with a conserved homolog, Bax, that accelerates programmed cell death. *Cell* **74**: 609–619

Pidgeon GP, Harmey JH, Kay E, DaCosta M, Redmond HP and Bouchier-Hayes DJ (1999) The role of endotoxin/lipopolysaccharide in surgically-induced tumour growth in a murine model of metastatic disease. *Br J Cancer* **81**: 1311–1317

Senger DR, Galli SJ, Dvorak AM, Peruzzi CA, Harvey VS and Dvorak HF (1983) Tumour cells secrete a vascular permeability factor that promotes accumulation of ascites fluid. *Science* **219**: 983–985

Shen B-Q, Lee DY, Gerber H-P, Keyt BA, Ferrara N and Zioncheck TF (1998) Homologous upregulation of KDR/Flk-1 receptor expression by vascular endothelial growth factor *in vitro. J Biol Chem* **273**: 29979–29985

Soker S, Takashima S, Miao HQ, Neufeld G and Klagsbrun M (1998) Neuropilin-1 is expressed by endothelial and tumour cells as an isoform-specific receptor for vascular endothelial growth factor. *Cell* **92**: 735–745

Thompson CB (1995) Apoptosis in the pathogenesis and treatment of disease. *Science* **267**: 1456–1462

 *© 2001 Cancer Research Campaign*
